# Dynamics of Ecosystem Services during Forest Transitions in Reventazón, Costa Rica

**DOI:** 10.1371/journal.pone.0158615

**Published:** 2016-07-08

**Authors:** Améline Vallet, Bruno Locatelli, Harold Levrel, Christian Brenes Pérez, Pablo Imbach, Natalia Estrada Carmona, Raphaël Manlay, Johan Oszwald

**Affiliations:** 1 CIRED, AgroParisTech, Cirad, CNRS, EHESS, Ecole des Ponts ParisTech, Université Paris-Saclay, Nogent-sur-Marne, France; 2 CIRAD, Research Unit on Forests and Societies, Montpellier, France; 3 CIFOR, Lima, Peru; 4 CATIE, Turrialba, Costa Rica; 5 Bioversity International, Montpellier, France; 6 AgroParisTech, Unité Mixte de Recherche Eco&Sols, Montpellier, France; 7 Université Rennes 2, Unité Mixte de Recherche CNRS 6554—LETG Rennes COSTEL, Rennes, France; Kerala Forest Research Institute, INDIA

## Abstract

The forest transition framework describes the temporal changes of forest areas with economic development. A first phase of forest contraction is followed by a second phase of expansion once a turning point is reached. This framework does not differentiate forest types or ecosystem services, and describes forests regardless of their contribution to human well-being. For several decades, deforestation in many tropical regions has degraded ecosystem services, such as watershed regulation, while increasing provisioning services from agriculture, for example, food. Forest transitions and expansion have been observed in some countries, but their consequences for ecosystem services are often unclear. We analyzed the implications of forest cover change on ecosystem services in Costa Rica, where a forest transition has been suggested. A review of literature and secondary data on forest and ecosystem services in Costa Rica indicated that forest transition might have led to an ecosystem services transition. We modeled and mapped the changes of selected ecosystem services in the upper part of the Reventazón watershed and analyzed how supply changed over time in order to identify possible transitions in ecosystem services. The modeled changes of ecosystem services is similar to the second phase of a forest transition but no turning point was identified, probably because of the limited temporal scope of the analysis. Trends of provisioning and regulating services and their tradeoffs were opposite in different spatial subunits of our study area, which highlights the importance of scale in the analysis of ecosystem services and forest transitions. The ecosystem services transition framework proposed in this study is useful for analyzing the temporal changes of ecosystem services and linking socio-economic drivers to ecosystem services demand at different scales.

## Introduction

Managing multiple ecosystem services (ES) across landscapes is challenging given that tradeoffs often occur in space and time [1–4] among bundles of multiple ES, including provisioning (i.e. products such as fibers, fuel and foods), regulating (e.g. climate, disease or water regulation) and cultural (recreation, education or heritage) services [5]. In contrast to the spatial dimensions of ES tradeoffs, the temporal dimension is relatively poorly studied [6,7] and recent studies have called for a better understanding of ES dynamics over time, their drivers and their implications for ES tradeoffs [7–13]. Historical ES analysis can help explain current ES levels, identify landscape management opportunities [14], and improve decision-making by providing scenarios needed to understand the impacts of socio-economic drivers on ES and to predict future ES [14,15].

The temporal changes of ES remains poorly understood. Only 11 out of 50 studies reviewed by Pagella and Sinclair [14] assessed past or future ES. Temporal ES dynamics are studied using economic valuation [16–18], historical land-cover data as ES proxies [19], paleoenvironmental records [11], literature and data review [10], and modeling with tools like InVEST [20–22] or with *ad hoc* models [23,24]. Few studies assess ES dynamics using biophysical models and local data that link ES changes to socio-economic drivers, including ES demand [10].

In comparison, forest-cover dynamics have been widely studied [25] and linked to socio-economic drivers, particularly in the forest transition framework (detailed in the next section) [26,27]. For example, in Costa Rica, after decades of deforestation, forest area is now considered stabilized or increasing in some parts of the country [28–30] due to reforestation and spontaneous regrowth, even though varying estimates make it difficult to confirm forest transition at the national scale [25,31].

Forest transition can have contrasting implications for the provision of multiple ES, depending on forest type and landscape management. For example, the recovery of regulating ES with forest expansion is debated [19,32]: in the second phase of the forest transition, forest expansion often results in improved regulating services but the expansion of certain types of forest plantations can also degrade water- and soil-related services [33,34].

This paper aims to analyze forest transition and the dynamics of ES in Costa Rica. We test the existence of an ES transition in the upper part of the Reventazón watershed in Costa Rica by assessing the variations of six ES in space and time from 1986 to 2008. We hypothesize that food provision increased in the early stages of development at the expense of regulating ES and that there was a recent inversion of this trend. The next section introduces the analytical framework, followed by a section presenting evidence of ES transition in Costa Rica from literature and secondary data. After a description of material and methods used for the modeling of ES, the changes of forest areas and ES are reported and discussed.

## Background and Analytical Framework

Given the importance of forests for biodiversity, water, timber and climate, forest dynamics have been widely studied [25], for example through the lens of the forest transition framework [26]. This framework describes two major stages in the development trajectories of countries or regions: first, population growth and increasing food demand lead to forest clearing for agriculture; second, agricultural intensification, urbanization, industrialization and the increasing scarcity of forest products lead to trend inversion and forest expansion [26,35]. Forest expands along two possible paths: the ‘economic development path’ (urbanization and industrialization create rural exoduses and land abandonment, while technological progress increases agricultural productivity and reduces demand for land); and the ‘forest scarcity path’ (scarcity and increasing prices of forest products induce private actors to plant trees and public decision makers to develop reforestation policies) [27,29,30,33–35].

Forest transitions have been documented in Europe and North America during the 19th and 20th centuries [27]. Some studies have focused on developing countries but with different degrees of evidence [25,36]: for example, the reversal is certain in Vietnam and likely in India, but more evidence is needed for Costa Rica [30,37,38]. The forest transition framework has been criticized, for overlooking differences in forest types (e.g. plantations or natural forests) and their corresponding ES [33,34]. ES can change without changes in forest areas, for example, from natural forests to plantations [39,40]. Forest expansion can occur through spontaneous regeneration, agroforestry, and mixed or monospecific plantations of exotic or native species, with different impacts on ES [41]. Thus, increasing forest areas are not always beneficial to water- and soil-related services or biodiversity [42,43].

**Fig 1:**
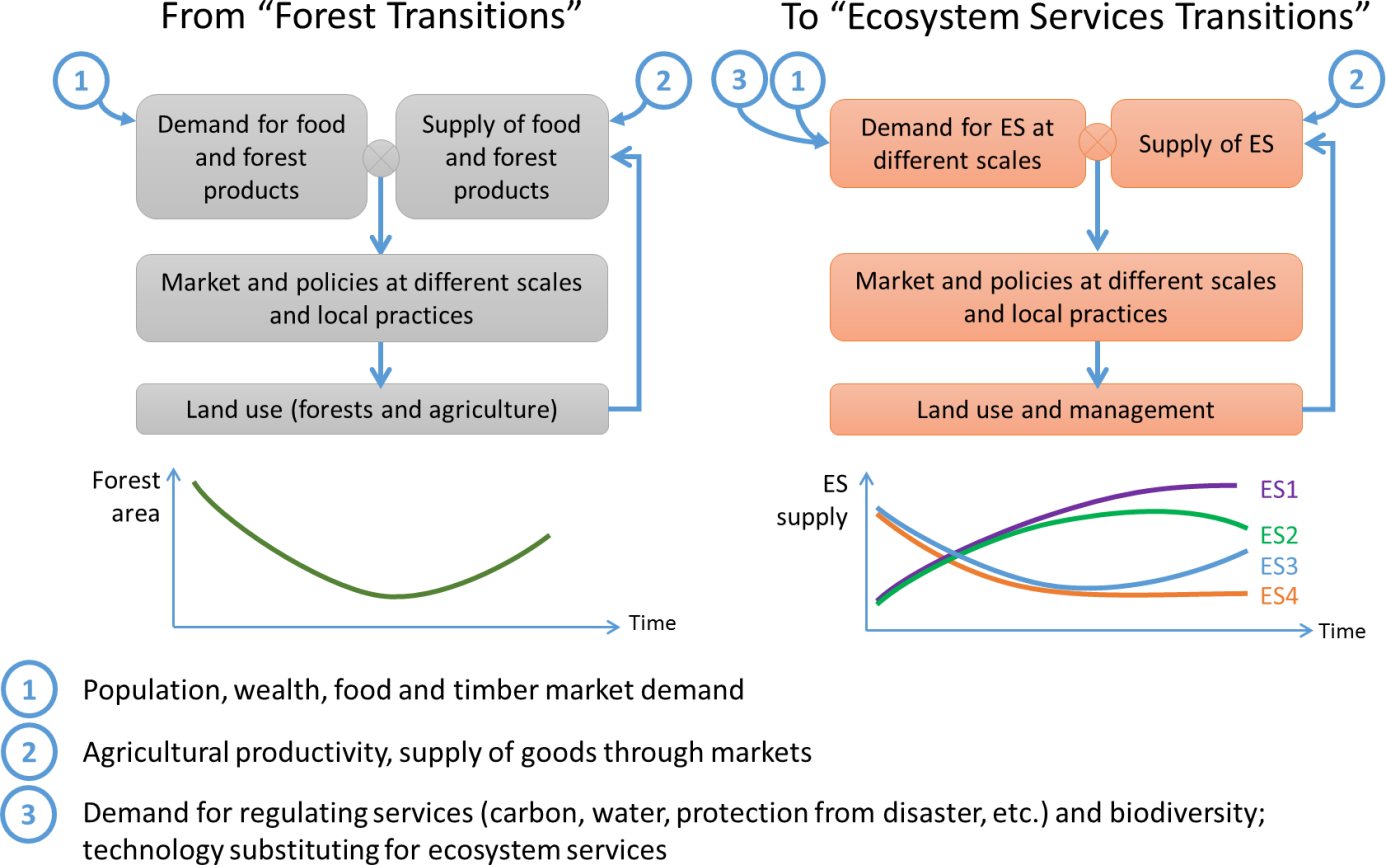
Forest transition and ES transition frameworks.

The forest transition framework can be extended to consider changes in ES (Fig 1). This ES transition framework considers diverse land covers and their management, including diverse forest types, their effect on ES and the tradeoffs between them. For example, provisioning ES from agriculture may increase in the first stage of the forest transition model, at the expense of other services. Trends in ES are much more difficult to depict for the right part of the curve, as agricultural provisioning ES can decrease or stay stable, forest provisioning ES can still decrease even though forest area increases (e.g. if forest policies restrict forest harvesting), and regulating or cultural ES can have contrasting variations depending on forest type. The framework also recognizes that changes in ES are driven by demand for ES at different scales, for example, the global demand for carbon sequestration through financial incentives for developing countries to reduce emissions from deforestation and forest degradation (REDD+) or local demand for hydrological services through plans for adaptation to climate change [44].

## Is There Evidence of a Transition of Ecosystem Services in Costa Rica?

In the last decades, major socio-economic changes have influenced land cover and ES in Costa Rica in general and in the Reventazón watershed in particular (see study site section). From the 1940s to the 1980s, Costa Rica experienced high rates of deforestation driven by population growth, national and international demand for beef, timber or crops, colonization policies and improved road infrastructure [28,36]. In the country as a whole, forest area decreased from 67% in 1940 to 32% in 1977 and 17% in 1983. In mountain and low mountain rainforests, such as in the Reventazón, deforestation remained low from the 1940s to the 1970s (around 0.3% per year) but increased strongly later on (up to 3.8% per year until the 1980s) [45]. While deforestation is associated with increased provisioning services (crops, timber, fodder for meat and milk), it reduced carbon stocks and hydrological services: erosion rates grew rapidly from the 1970s to the 1990s in the cultivated, erodible and steep soils of the Reventazón [46] increasing costs for cleaning hydroelectric dams [47].

From the 1980s to 2000s, economic transformations occurred that pushed smallholders to diversify their activities [48]. The tourism sector increased steadily, with 10% more tourists each year from 1986 to 2000 and visits to protected areas increasing by 12% per year between 1982 and 1992 [49]. In 1994, tourism became the largest source of foreign exchange for Costa Rica, which was moving from an agrarian to a service economy [50]. In some areas, ecotourism opportunities pushed farmers to abandon agriculture and to restore forests for their new economic value [51]. Investments in real estate by foreign nature-lovers also had a significant impact on forest conservation and restoration [29].

During the same period, environmental and forest policies progressively changed in Costa Rica. Policies emerged in the 1980s for incentivizing reforestation and forest management on private lands and the export of logs was banned, but with limited success [28,50]. In 1996, a new forestry law restricted timber extraction and established a program of payments for environmental services (PES) [52]. Nature-related policies also involved the creation of national parks. Since national parks were legally created in 1969, areas under various kinds of protection have expanded and now cover around 25% of the national territory [48,50]. In the Reventazón watershed, the large Tapanti National Park was created in 1982. In addition, more than 80% of Cerros de la Carpintera, a protected area created in 1976, has now been reforested following widespread deforestation documented in 1960 [53].

Land-use decisions in the Reventazón watershed have been sometimes driven by the demand for hydrological ES: for example, ICE (Instituto Costariciense de Electricidad), a major Costa Rican hydroelectric company was involved in the creation of national parks upstream of hydroelectric plants [54]. More PES have been delivered to watersheds with actual or planned hydroelectric dams than to all other watersheds [55]. A recently established water fee will increase PES targeted at the conservation of hydrological services [56]. In addition, carbon sequestration has motivated new plantations and forest conservation in the area [57]. For example, the Pax Natura Foundation developed a carbon project for reducing deforestation and the Klinki Forestry project reforested pastures and marginal farmland with the support of voluntary carbon markets [58]. Although these projects may ultimately affect several thousands of hectares, their current contribution is limited.

Thus, in the 1990s, forest area trends in Costa Rica began to reverse, as a consequence of economic transformation and new environmental policies [31]. Even if forest degradation has continued [50], forest area is now considered to have stabilized or be increasing in the some parts of the country [28–30]. Estimates vary making it difficult to confirm forest transition at the national scale [25,31].

**Fig 2:**
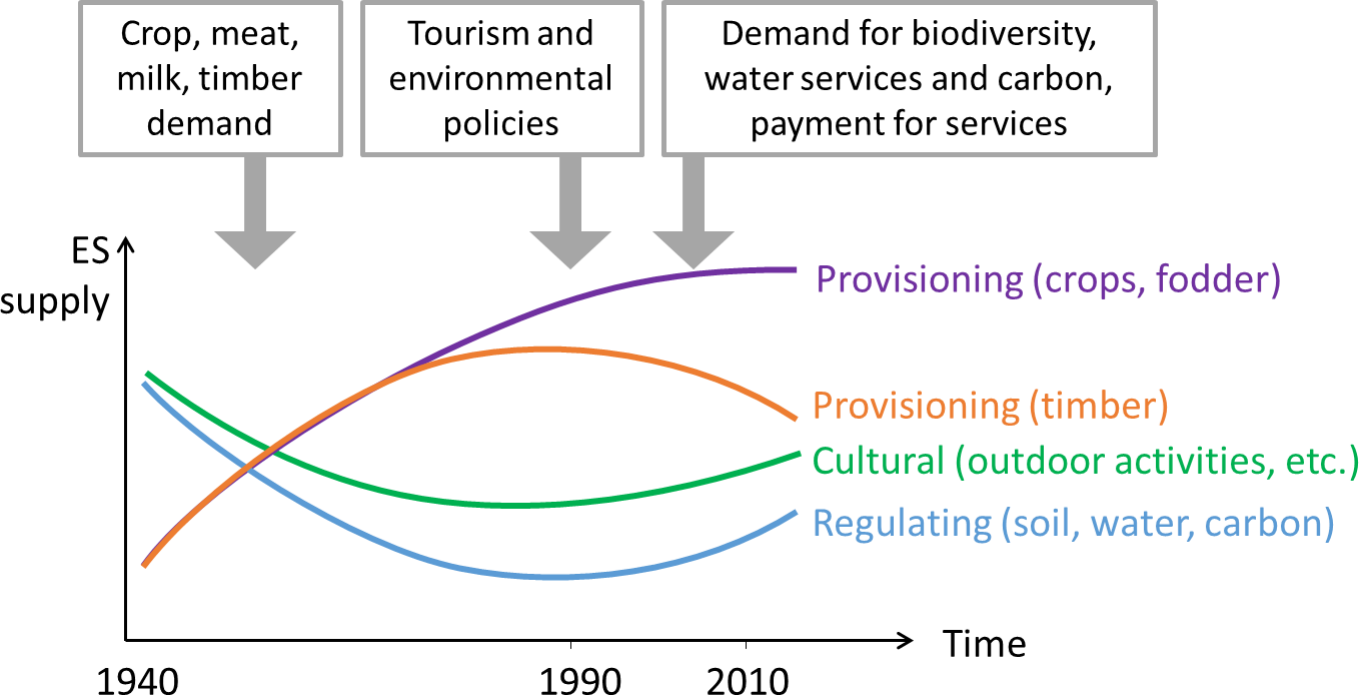
Simplified model of ES transition in the upper part of the Reventazón watershed as suggested by existing literature and databases.

Existing literature suggests an ES transition in our study site (Fig 2), even though some trends are still nascent and uncertain, particularly for provisioning services from agriculture. The production of the most represented crops in our study site (coffee and ornamental plants) has declined slightly since the early 2000s (-0.5%/yr), after two decades of growth (+2.2%/yr), while the production of dairy products has increased since the early 2000s (+3%/yr) [49]. There is no measurement of changes in soil erosion at the watershed scale or in agricultural areas, but forest regeneration in high slope and in cloud forest areas is likely to have increased the supply of soil- and water-related services, as well as carbon sequestration [54]. Similarly, forest regeneration and conservation have likely increased or protected services related to outdoor activities (animal watching, white water sports, etc.) as well as scenic beauty and heritage value associated with pristine forests by most tourists [59]. The production of timber does not show a clear trend in Costa Rica since the 1970s [49], but it now comes mainly from plantations, which are rare in our study site compared to northeastern and northwestern Costa Rica [60]. For this reason, the supply of timber is likely to have decreased in the upper Reventazón watershed. While the demand for provisioning services was a main driver of changes in landscapes and economic services from the 1940s to the 1980s, current changes are also driven by demand for regulating and cultural services related to water, carbon and tourism.

## Study Site

**Fig 3:**
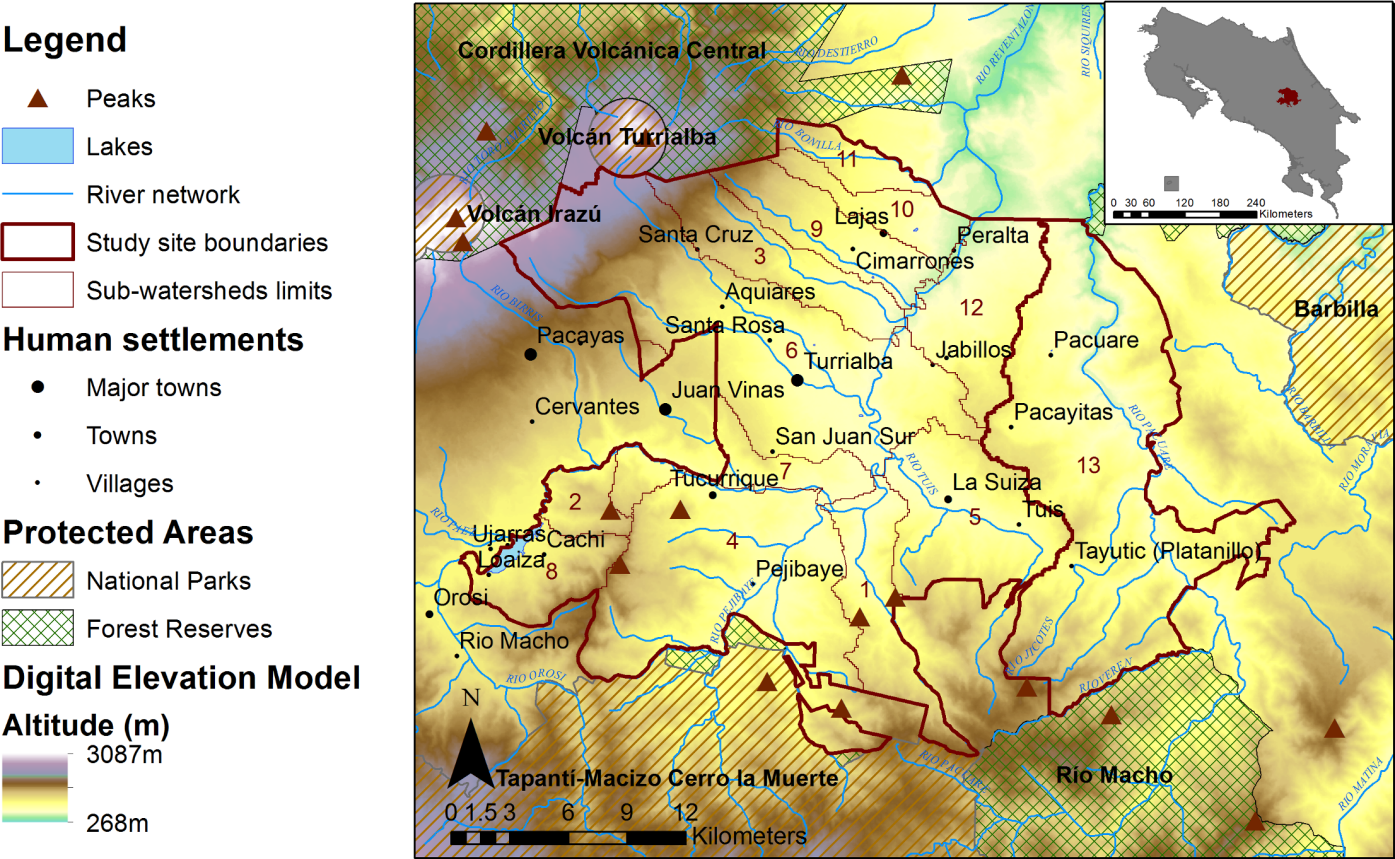
Location of the study site in Costa Rica.

The Volcanic Central Talamanca Biological Corridor is located on the Caribbean slopes of the central volcanic mountain range of Costa Rica (approximately centered around 9.87°N 83.63°W) and hosts the upper parts of two major rivers, Reventazón and Pacuare, whose entire watersheds represent 8% of the country area (Fig 3, with data from [60] and [61]). The site covers an area of 740 km^2^ (1.4% of Costa Rica) [62]. The topography is mountainous and elevation ranges from 268 m to 3087 m above sea level near the Irazú volcano. Climate is tropical humid with average rainfall between 1500 and 7000 mm/year (depending on elevation), irregularly distributed throughout the year with a peak of intensity between November and December [63]. According to Holdridge’s life zones, a gradient from premontane to montane altitudinal belt can be observed in the study site, with premontane wet and rain forests occupying a vast area in the lowlands (respectively 55 and 21% of the total study area); while montane and lower montane wet and rain forests are restricted to high mountains in the northwest and south of the study site [60,64]. Andept inceptisols and humult ultisols are the most common soil types in the study site [65,66]. They are characteristic of humid-tropical volcanic mountains and are rich in organic matter.

Forests are the most extensive land cover. Different types of forest ecosystems can be distinguished according to management, humidity and altitude: (1) wet and rain old forests (including secondary forest of 20 to 30 years old and patches of primary forest in remote areas; (2) “Charrales” or 3–10 years young secondary forests where thorny plants and bushes are abundant; (3) old forest plantations mostly dominated by Eucalyptus (planted approximatively in the 1980s by private landowners) [67]. In the rest of the article, “forests” refer to both natural and planted ecosystems, unless more details are given (old, young, or planted forests). This precision is particularly important when analyzing changes in forest covers (reforestation and deforestation processes) through the lens of forest transition [68]. Other main land covers are crops (vegetables, ornamentals, coffee, sugarcane) and pastures for dairy or meat production [53,69].”

The most important economic activities of the 80,000 inhabitants (about 1.7% of national population) are agriculture, cattle farming, industry, trade and tourism [70]. The Reventazón watershed is highly strategic for the national economy, as it represents 25% of national hydropower, 30% of milk and meat production, 85% of potato and onion production, and 23% of flower and ornamental plant exports [53].

The study site was selected because of its relevance for the production of multiple ecosystem services (agricultural production, carbon sequestration, cattle farming, tourism, hydroelectricity production), the shifts in land-cover change drivers (an initial strong demand for agriculture and forest products driving deforestation, gradually replaced by incentives for reforestation and nature protection and touristic development) and the availability of fine-scale and reliable land-cover data at various dates. The temporal scope of the study was conditioned by existing land-cover maps at different dates, which were developed using a homogenous methodology for land -over classification over the 1986–2008 period [62].

## Materials and Methods

We assessed the changes of six ES from 1986 to 2008. We selected one provisioning ES (agricultural production) and five regulating services: carbon storage (capacity to store carbon and mitigate climate change), water yield (quantity of water released), nitrogen and phosphorus retention (contribution of plants and soil to nutrient retention from runoff) and sediment retention (capacity to prevent soil erosion). These ES are particularly relevant for the study area, given its agricultural potential, its propensity to soil erosion and the economic utility of water-related activities, such as hydropower production.

Agricultural production was assessed by the total added value of goods produced on agricultural lands, calculated from prices and yields for each agricultural product (S1 File). Regulating ES were modeled with the InVEST (Integrated Valuation of Ecosystem Services and Tradeoffs) tool version 3.1, an open-source program developed by the Natural Capital Project [71]. InVEST consists of a set of deterministic models that estimate the supply and economic value of ES given land-cover maps and related biophysical and economic data [3,71–74]. ES are quantified through coefficient tables for each land cover associated with models of flux of water, nutrient and sediment through the landscape [72]. The InVEST release we used includes three supporting ES and fifteen final ES provided by marine, fresh water and terrestrial systems [71]. In this study, we only used a small subset of services modeled by InVEST, and following [3] we reported ES in biophysical terms exclusively.

In the carbon storage model, each land cover was associated with a total carbon stock per unit of area (S1 File). The four water- and soil-related ES were assessed by InVEST with a hydrological model using multiple spatial data (Table 1) and land-cover coefficients (S1 File). Following InVEST recommendations [71], land-cover coefficients (e.g. carbon stored in each land-cover type) were determined with a three-tier literature review: local data were searched and used preferentially but, if unavailable, they were substituted with national data, which, if also unavailable, were substituted with global data (S1 File). Water yield was calculated as the difference between precipitation and evapotranspiration, estimated from a reference evapotranspiration value adjusted for different land covers [71]. The nutrient retention model assessed nutrient exports from one pixel as a function of export coefficients by land-cover types, water runoff and the cumulative nutrient charge of neighboring pixels. Sediment retention was calculated from a soil-loss estimate (with the Universal Equation of Soil Loss [75]).

**Table 1:** Spatial data used to assess ES or to present the results of ES assessments.

ES	Variable	Data	Reference
All ES	Land cover	Exisiting land-cover maps at 30m resolution for 1986, 1996, 2001 and 2008, from satellite images (ASTER and Landsat) and orthorectified photographs	[62]
	Administrative boundaries, road network, river network, populated places	Base maps from the Digital Atlas of Costa Rica	[60]
	Sub-watershed limits	Delineated from Digital Elevation Models and river network shapefile using ArcHydro tools in ArcGIS	[76]
All water- and soil-related ES	Precipitation	Average annual precipitations (1950–2000) from WorldClim (1km resolution)	[77]
	Topography	30m resolution Digital Elevation Model from the ASTER GDEM project	[61]
	Soil depth and Available water capacity	Soil parameters from FAO database	[78]
Water yield	Reference annual evapotranspiration	Global Potential Evapo-Transpiration high-resolution database by CGIAR-CSI (1km resolution)	[79]
Sediment retention	Rainfall erosivity	Spatial extrapolation of measurement of storm energy and intensity in weather stations	[69]
	Soil erodibility	Soil parameters and map from FAO database	[69]

To analyze and compare ES changes, ES estimated levels were log-transformed (if they had a skewed distribution) and standardized with a Z-score normalization (resulting in values with a mean of 0 and a standard deviation of 1, See S2 File). To highlight different dynamics within the study area, we defined three groups of sub-watersheds based on changes in forested areas from 1986 to 2008: large increase (in more than 3% of the area, a threshold defined arbitrarily as the 80% quantile of the distribution of the absolute values of forest area changes), moderate increase (in less than 3% of the area), and decrease or no change (in less than 3% of the area). To analyze changes in forests area, we considered all forest ecosystems described previously: old, young and planted forests. The k-means algorithm was used to cluster the 13 sub-watersheds according to the changes of ES observed in these sub-watersheds between 1986 and 2008. All analysis used R software [80] and the raster package [81].

## Results

**Fig 4:**
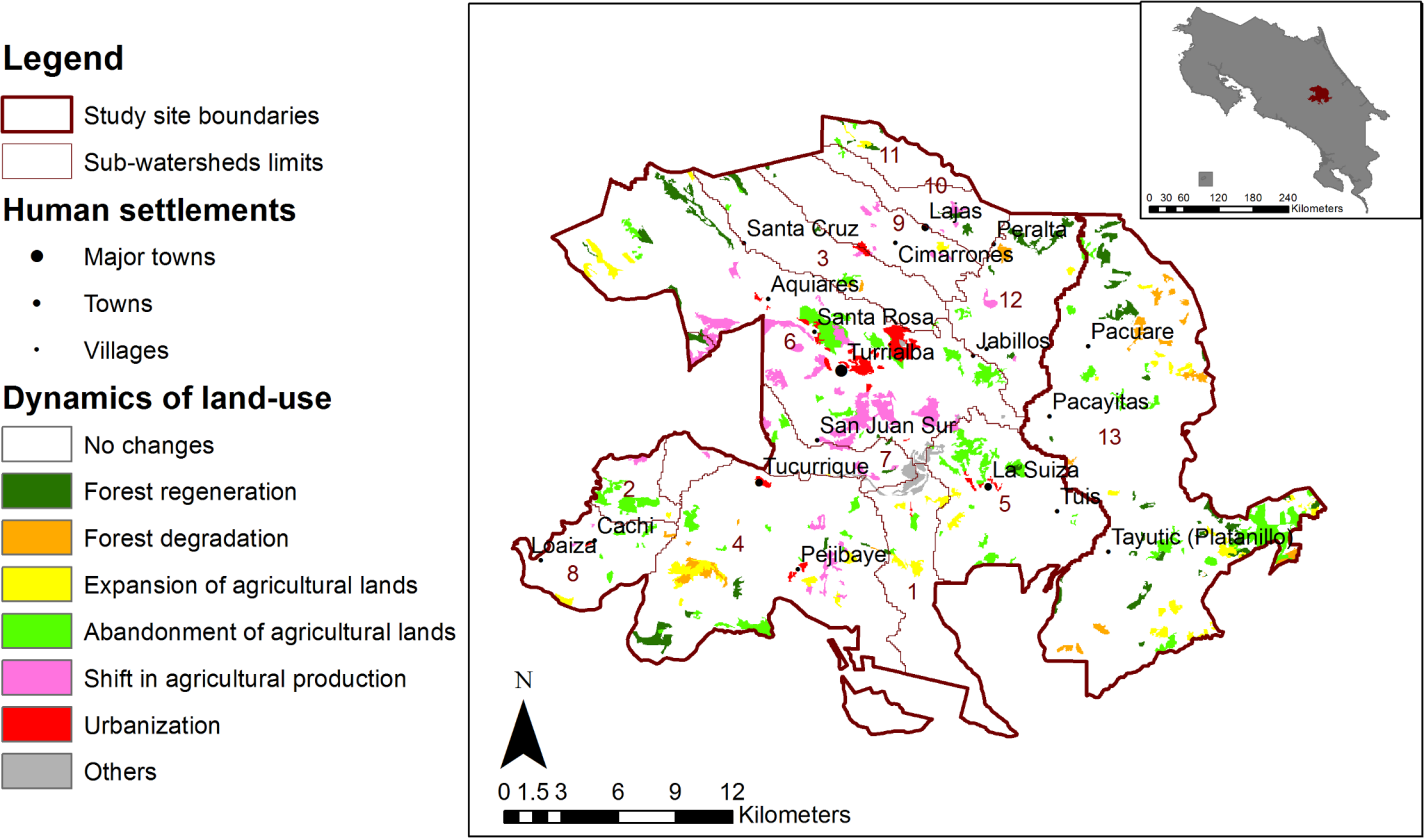
Land-cover changes between 1986 and 2008 in the study site (data from [62]). The gray area in the center of the map represents the Angostura reservoir built between 1996 and 2001. Numbers identify the 13 sub-watersheds.

Land-cover changes occurred in a small and decreasing part of the area (7.4% in 1986–1996 and 2.6% in 2001–2008), where old forests and crops expanded, while pasture and coffee plantations shrank (See S3 File). Six major land-cover changes occurred (Fig 4), presented in decreasing order of area: (1) from agriculture to young forests (following abandonment of coffee plantations and pastures); (2) from young to old forests (forest regeneration); (3) from old or young forests to agriculture (expansion of pastures, coffee and crops); (4) shift in agricultural production (e.g. coffee to horticulture, pasture to sugarcane); (5) from old to young forests (forest degradation); (6) urbanization. The first two classes represented more than 40% of the observed changes and 60% in the last period 2001–2008. Urbanization, abandonment of agricultural lands and shifts in agricultural production occurred close to roads, while forest degradation took place further from roads. Forest regeneration occurred more on steep slopes, while shifts in agricultural production and urbanization happened more in flat areas (see S5 File for more details on models of land-cover changes).

**Fig 5:**
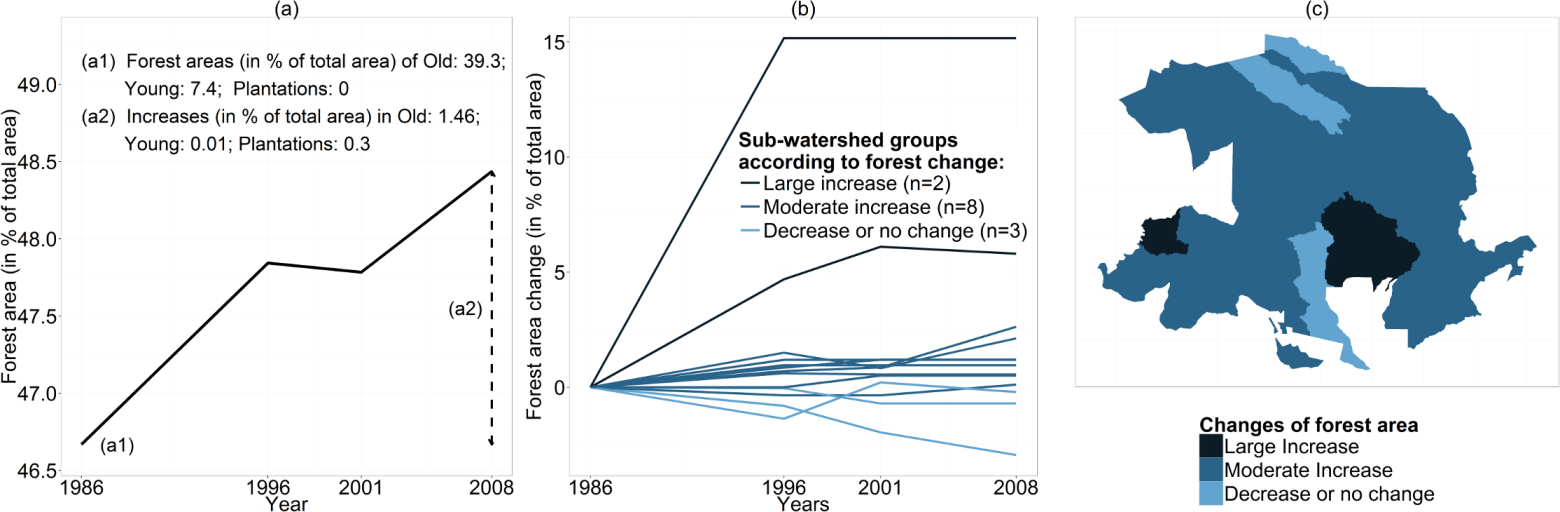
Changes of forest area from 1986 to 2008. (a) In the whole area; (b) in each the 13 sub-watersheds; (c) location of sub-watersheds in the three groups defined by large increase in forest area (in more than 3% of the area, n = 2), moderate increase (n = 8) and decrease or no change (n = 3).

The results showed that forest areas increased from 46.7% to 48.5% of the study area between 1986 and 2008, mostly through old and planted forests (Fig 5A). There were large differences among the 13 sub-watersheds: forests expanded in 15% of the area in sub-watershed 2, while they shrank by 3% in sub-watershed 1 (Fig 5B). There were more sub-watersheds with moderate increases in forest areas between 1986 and 2008 than large increases (sub-watersheds 2 and 5) and decrease (sub-watersheds 1, 9 and 11) (Fig 5B and 5C). Five sub-watersheds had non-monotonic changes of forest areas but only sub-watershed four showed changes similar the forest transition framework (decreasing then increasing forest area) (Fig 5B).

**Fig 6:**
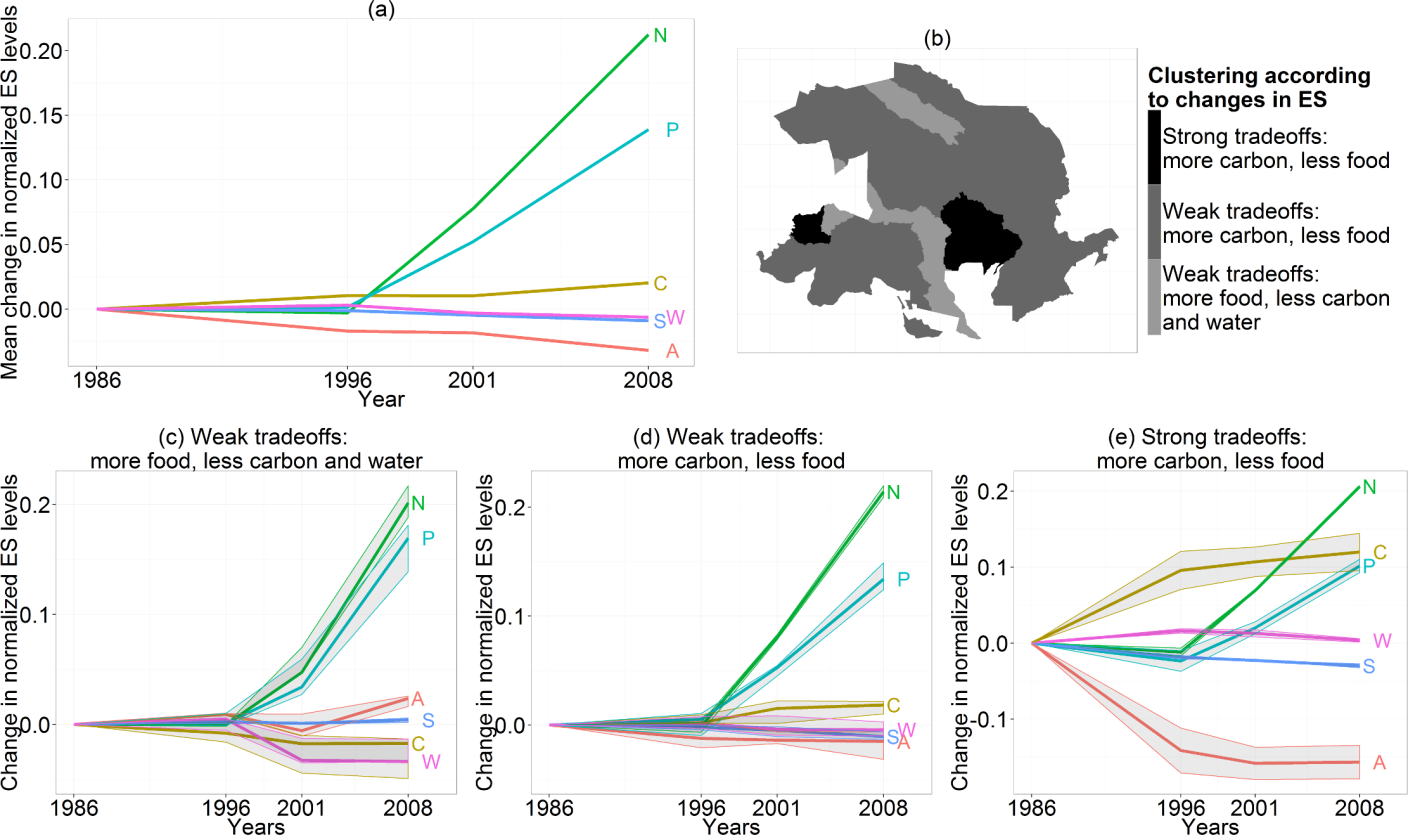
Changes of ecosystem services from 1986 to 2008. (a) Mean changes in the levels of the six selected ES. (A: agricultural production, C: carbon, N: nitrogen retention, P: phosphorus retention, S: sediment retention, W: water yield); (b) location of sub-watersheds in the three clusters defined by ES changes: (1) “Weak tradeoffs: more food, less carbon and water” (n = 3); (2) “Weak tradeoffs: more carbon, less food” (n = 8); (3) “Strong tradeoffs: more carbon, less food” (n = 2); (c-e): changes in ES levels in the three clusters of sub-watersheds (lines represent the median values of the group elements, ribbons represent the interquartile range).

Mean values of carbon sequestration and agricultural production over the whole study area showed clear tradeoffs, with carbon increasing over time and agricultural production decreasing (Fig 6A). Nitrogen and phosphorus retention increased strongly and other ES had limited changes (Fig 6A). Only water yield had a non-monotonic change (first an increase followed by two time periods of decrease). Sub-watersheds belonged to three clusters described by the tradeoffs between agricultural goods, carbon and water, given that nitrogen and phosphorus retention increased everywhere regardless of sub-watershed (S4 File).

The changes of ES and their tradeoffs were similar in the cluster “Weak tradeoffs: more carbon, less food” and in the overall study area (Fig 6D). The two sub-watersheds of the cluster “Strong tradeoffs: more carbon, less food” showed a stronger increase in carbon sequestration and a decrease in agricultural production (Fig 6E) corresponding to the sub-watersheds with large increases in forest areas (Figs 6B and 5C). The three sub-watersheds of the cluster “Weak tradeoffs: more food, less carbon and water” followed opposite trends, with increasing agricultural production and decreasing carbon sequestration and water yield (Fig 6C), two of them were sub-watersheds with decreasing forest areas (Figs 6B and 5C).

## Discussion

Our land-cover change analysis showed no clear evidence of a forest transition in the study area, as forest areas were steadily increasing during the period of analysis and no inversion of forest area trends was observed, as in another study about forest trends in Costa Rica [25]. Given that our study area experienced deforestation before the 1980s, the current forest trends may suggest that the turning point occurred before the beginning of our period of analysis (i.e. before 1986) and that the area is currently experiencing a post-transition regime. A major limitation of our work and, more generally, of such historical studies is the short time period over which statistical data and land-cover maps are available [25]. Another technical limit is the accuracy of remote-sensing reflectance measurements that hardly differentiate between agroforests and plantations, leading to error in classification of land-cover areas [62].

The forest transition framework has often been applied at national scales [36]. However, forest area trends depend on the scale at which they are observed, highlighting the need to conduct multiple-scale assessments [36,82]. As in our study, a scale effect was observed between national and subnational levels in Puerto-Rico, with a national net reforestation that masked the loss of primary and secondary forest at the subnational scales in some areas [82]. In our study, only one sub-watershed followed the forest transition model (with forest contraction followed by expansion) while most others had monotonic increases or decreases in forest areas. At the scale of Costa Rica, forest transition is still discussed [25,30], which may be explained by the fact that different regions are at different stages of forest transition: during, after the turning point (as may be the case of our whole study area) or before.

Forest expansion occurred mainly through abandonment of agricultural lands (as also observed in Costa Rica by Arroyo-Mora et al. [83]) and forest regeneration (from young to old forests) rather than forest plantations, which have expanded in other places in Central and South America where forest areas have increased [19,33]. This could be explained by different underlying drivers of the forest transition: economic changes [48,83] and PES [48,84] may have led to agricultural land abandonment and forest regeneration in the case of our study area while forest product scarcity may have led to forest plantations elsewhere [31,85,86]. Further research should investigate the spatial effects of drivers on forest regrowth, for example, whether reforestation occurs in areas abandoned because of their low profitability [82] or whether environmental policies and the creation of a biological corridor project in our study site influenced forest expansion.

No clear ES transition was observed in our quantitative analysis, probably because of the short time period allowed by the data. The review of literature and databases suggested that, since the 1990s, provisioning services have been decreasing and regulating services have been increasing. Our modeling results showed these trends for agricultural products and for carbon sequestration over our whole study site, but we could not identify the point at which these trends started. For this reason, it is important to combine quantitative assessment with qualitative analysis of ES changes since the latter can help identifying transitions that do not appear through the former. All sub-watersheds showed an increase in nitrogen and phosphorus retention that resulted from two distinct mechanisms: (1) an increase in nutrient retention capacity by forests in the sub-watersheds with increasing forest cover and (2) an increase in nutrient loads in the sub-watersheds with agricultural expansion or shifts toward highly fertilized crops (from coffee to horticulture and from pasture to sugarcane, see S1 File). The ES dynamics of some sub-watersheds followed the trends of the first phase of the ES transition (more goods, less regulating services) while others showed opposite ES trend, which is expected in the second phase of the forest transition (fewer goods, more regulating services). Even though we could not observe a turning point within the study area as a whole, the analysis at the sub-watershed scale identified different ES dynamics and tradeoffs representing pre- and post-transition regimes.

This specialization of landscape (or land sparing) for the production of specific bundles of ES was also observed in Canada [7] and similarly led to the concentration of agricultural production in some areas while forests regenerated elsewhere. In Argentina, the temporal dynamics of ES from 1956 to 2005 also presented a strong variability between the 21 eco-regions [24]. This spatial heterogeneity of ES often results from the spatial variability of ES demand, based on socio-economic characteristics [7].

Like other studies [7,10], our research showed that changes in ES also reveal changes in drivers. While we did not analyze drivers of ES changes in detail, the literature review on Costa Rica suggested that economic transformations and environmental policies have driven an ES transition since the 1990s in the country. While the demand for provisioning services was a main driver of changes in landscapes and economic services from the 1940s to the 1980s, current changes are driven by demand for regulating services related to water and carbon as well as demand for cultural services and tourism. In Spain, similar changes have been observed. The demand for ES has changed over the last 60 years: demand for local provisioning ES (particularly food) has dwindled because of competitive international food prices while national and international demand for cultural and regulating services has increased [10]. In Québec [7], attractive market prices and regional subsidies for corn production have encouraged agricultural specialization. Further research could focus on analyzing the drivers of ES dynamics linked to ES demand from local to global levels. Different tradeoffs between ES could be highlighted in different sub-watersheds and over different time periods in our case study. Similarly, in Québec, tradeoffs and synergies between ES changed over time and could even be inversed: animal production and cultural services shifted from conflicting ES to synergetic ES, mostly due to the conversion of traditional outdoor breeding to confined breeding [7]. In our analysis, tradeoffs occurred mainly between agricultural production and carbon sequestration. Nitrogen and phosphorus retention showed a clear synergy, while other regulating services had less clear relationships with other services. This could be due to the limitations of the InVEST model, which has a simplified representation of water yield and sediment or nutrient retention [21,71,87,88]. Water- and soil-related services are complex and may require more sophisticated approaches to analyze ES interactions and the mechanisms behind them in space and time [89]. As in our study, an historical perspective in Québec [7] showed a significant and consistent tradeoff between crop production and carbon storage over time, as well as no clear pattern of interaction between hydrological services (flood control) and other services. Using static approaches, several studies have also showed the existence of tradeoffs between production services and carbon storage [4,90,91] even though other authors concluded that such patterns of interaction between provisioning and regulating services should not be generalized without caution [92].

Another limitation of this study is the poor consideration of biodiversity, which is a critical component of mosaic landscapes, and should be better integrated into the analysis of forest and ES transitions [93–95]. Not considering biodiversity could lead to overlook tradeoffs between ES it sustains [90,96]. For example, the demand for timber or carbon sequestration as ES can lead to the expansion of monoculture plantations with exotic species, which can affect soil biodiversity and processes or biodiversity at landscape level [68]. Biodiversity could be integrated in our framework as a part of ecosystem processes or services (e.g., pest regulation, spiritual values, and goods produced from genetic diversity) [97]. The ES transition framework we explored in this study is useful to account for the demand-driven nature of temporal ES dynamics. It links socio-economic drivers at different scales to the levels of ES in different time periods.

More research is needed to refine and test this framework and to make it more operational. Further research could help to (1) better understand ES transitions, for example by classifying transitions depending on drivers, ES tradeoffs and magnitude or velocity of ES changes, (2) describe scale effects on transitions, (3) link non-spatial drivers to spatially heterogeneous ES changes, (4) understand the feedback effects of ES levels on ES demand and (5) analyze the temporal and spatial lags between changes in demand for ES and their effect on ES dynamics. Given that the rate of forest recovery is considerably slower than the speed of deforestation, future research could specifically focus on comparing ES time lags before and after forest transition. There is also a need for further analyses of the implications of forest transitions for ES in different contexts and study sites, before, during and after transitions.

## Conclusions

The objective of this study was to analyze land cover and ES in space and time in an area in Costa Rica where forest transition has been suggested. We introduced an analytical framework to link the dynamics of ES to forest transitions and socio-economic drivers at different scales. The study did not find evidence of a forest transition or an ES transition at the scale of the whole study area but the results suggested that the turning point of the transition may have occurred before the beginning of our study period. Some trends are, however, only nascent, particularly for some regulating services like soil and water conservation. At the scale of sub-watersheds, ES trends are diverse and can be similar or opposite to the trends observed at the whole study area scale, which highlights the importance of scale in the analysis of forest transitions and ES transitions.

## Supporting Information

S1 File Parameters used in ES modeling. (PDF)

S2 File Transformation of ES variables. (PDF)

S3 File Details on land-cover changes. (PDF)

S4 File Results of the sub-watershed cluster analysis. (PDF)

S5 File Linear models of land-cover changes. (PDF)
