# Supplementary material for: Dynamics of Ecosystem Services during Forest Transitions in Reventazón, Costa Rica
Source: PLoS One. 2016 Jul 8;11(7):e0158615. doi: 10.1371/journal.pone.0158615 (PMC4938395; doi:10.1371/journal.pone.0158615)
Supplement: S1 File — (PDF) [file pone.0158615.s001.pdf]

S1. Parameters used in ES modeling.

Table 1: Parameters used in InVEST models for water yield and nutrient retention. Parameters were determined with a literature review (references are indicated by numbers in brackets below the values, full references are provided after Table 2). LU_code: code of each land use; LU_desc: description of each land use; LU_vg: code that determines which evapotranspiration equation InVEST should use regarding vegetation presence/absence; root_depth: maximum root depth in mm; Kc: plant evapotranspiration coefficient; usle_c: cover-management factor of the Universal Soil Loss Equation; usle_p: support practice of the Universal Soil Loss Equation; load_n: nitrogen loading in $kg.ha^{-1}.yr^{-1}$; load_p: phosphorus loading in $kg.ha^{-1}.yr^{-1}$; eff_n: nitrogen vegetation filtering value per pixel; eff_p: phosphorus vegetation filtering value per pixel.

LU_ code	LU_desc	LU_vg	root_depth	Kc	usle_c	usle_p	load_n	load_p	eff_n	eff_p
1	Old forests	1	4157 [1–7]	0.97 [3,8–10]	0.003 [11]	1	2.89 [2,4,12–16]	0.077 [2,4,13,14,17]	0.51 [2–4,18]	0.51 [2–4,18]
2	Pastures	1	683 [6,7,19]	0.94 [3,8–10]	0.017 [11,20,21]	1	5.855 [4,14–16,22]	0.583 [4,14,17]	0.36 [3,4,18]	0.36 [3,4,18]
3	Young forests	1	2433 [2–4,6]	0.78 [3,9,10]	0.015 [11,20]	1	3.267 [2–4]	0.171 [2–4]	0.41 [2–4,18]	0.41 [2–4,18]
4	Sugarcane plantations	1	1308 [5,7,19]	0.85 [9,19]	0.185 [11,23–25]	1	13.361 [16,26–30]	4.195 [16,26,30]	Same as crops	
5	Coffee plantations	1	1167 [3,6,31]	0.85 [8–10,32]	0.058 [11]	1	3.298 [12,22,33]	0.096 [2,22]	0.25 [2]	0.25 [2]
6	Urban areas	0	340 [2–4,6]	0.28 [3,10]	0.011 [11]	1	6.316 [2,4,13–15]	1.818 [2,4,13–15]	0.03 [2–4,18]	0.03 [2–4,18]
7	Water bodies	0	128 [3,4,6]	1.15 [3,9,10,34]	0 [11,20]	1	0 [2–4,35]	0 [2–4]	0.02 [3,4,18]	0.02 [3,4,18]
8	Crops	1	570 [6,19]	0.82 [3,9,10]	0.381 [11]	1	11.925 [2,4,13,14,36]	1.14 [2,4,13,14,36]	0.13 [2–4]	0.13 [2–4]
9	Bare soil	0	53 [2–4]	0.25 [3,9,10]	0.883 [11,20,37]	1	0.035 [2,4]	0.001 [2–4]	0.05 [2–4]	0.05 [2–4]
10	Forest plantations	Same as forests								
11	Crops under net	Same as crops								
12	Rural areas planned for urbanization	Average of old forests, pastures and forest plantations								

Table 2: Parameters used in InVEST models for carbon sequestration and for ad hoc modeling of agricultural production. Parameters were determined with a literature review (references are indicated by numbers in brackets below the values, full references are provided after Table 2). LU_code: code of each land use; LU_desc: description of each land use; LU_vg: code that determine which evapotranspiration equation InVEST should use regarding vegetation presence/absence; C_above: amount of carbon stored in aboveground biomass in $Mg.ha^{-1}$; C_below: amount of carbon stored in belowground biomass in $Mg.ha^{-1}$; C_soil: amount of carbon stored in soil in $Mg.ha^{-1}$; C_dead: amount of carbon stored in dead organic matter in $Mg.ha^{-1}$; Agri_prod: Total added value (in Costa Rican colon) of goods produced on agricultural lands in $CRC.ha^{-1}$.

LU_code	LU_desc	LU_vg	C_above	C_below	C_soil	C_dead	Agri_prod
1	Old forests	1	73 [38]	18 [38]	209 [38]	9 [38]	0
2	Pastures	1	2 [39–44]	2 [41,42,44]	127 [39,41–44]	0	396668 [45,46]
3	Young forests	1	20 [38]	5 [38]	208 [38]	4 [38]	0
4	Sugarcane plantations	1	12 [47,48]	2 [47,48]	134 [49]	3 [47]	758430 [50,51]
5	Coffee plantations	1	14 [40,52]	4 [52]	124 [40,52]	2 [52]	297663 [50,51]
6	Urban areas	0	0 [3]	0 [3]	0 [3]	0 [3]	0
7	Water bodies	0	0 [3]	0 [3]	0 [3]	0 [3]	0
8	Crops	1	0 [53]	0 [53]	60 [53]	0 [53]	7882732 [46,50,51]
9	Bare soil	0	0 [3]	0 [3]	60 [53]	0 [3]	0
10	Forest plantations	1	62 [54,55]	14 [54,55]	114 [54,55]	5 [54,55]	0
11	Crops under net	Same as crops					
12	Rural areas planned for urbanization	Average of old forests, pastures and forest plantations					

References for Tables 1 and 2

1. Canadell J, Jackson RB, Ehleringer JB, Mooney HA, Sala OE, Schulze E-D. Maximum rooting depth of vegetation types at the global scale. Oecologia. 1996;108: 583–595. doi:10.1007/BF00329030

2. Bhagabati N, Barano T, Conte M, Ennaanay D, Hadian O, McKenzie E, et al. A Green Vision for Sumatra: Using ecosystem services information to make recommendations for sustainable land use planning at the provice and district level [Internet]. Indonesia: Natural Capital Project, WWF-US, and WWF-Indonesia; 2012. Available: http://www.naturalcapitalproject.org/where/sumatra.html

3. Tallis, HT, Ricketts, T, Guerry, AD, Wood, SA, Sharp, R, Nelson, E, et al. InVEST 3.0.0 User's Guide Integrated Valuation of Environmental Services and Tradeoffs. Stanford, USA: The Natural Capital Project; 2013.

4. Leh MDK, Matlock MD, Cummings EC, Nalley LL. Quantifying and mapping multiple ecosystem services change in West Africa. Agric Ecosyst Environ. 2013;165: 6–18. doi:10.1016/j.agee.2012.12.001

5. Schosinsky G. Cálculo de la recarga potencial de acuíferos mediante un balance hídrico de suelos. Rev Geológica América Cent. 2006; 13–30.

6. Faustino Manco J, Benegas Negri L, Gómez M, Watler Reyes WJ, Ney Rios J, Oduber Rivera J, et al. Caracterización, diagnóstico, línea base y zonificación territorial de la cuenca del Río Jesús María [Internet]. Turrialba, Costa Rica: CATIE-FONAFIFO; 2011. Available: http://www.fonafifo.go.cr/documentacion/biblioteca/consultorias_investigaciones/ce_psa_005.pdf

7. Blanco-Rojas, H. Áreas de recarga hídrica de la parte media-alta de las microcuencas Palo, Marín y San Rafaelito, San Carlos, Costa Rica. Cuad Investig UNED. 2010; 181–204.

8. Ramírez Builes V, Jaramillo Robledo A. Balances de energía asociados a los cambios de cobertura en la Zona Andina colombiana. Cenicafé. 2009;60(3): 199–210.

9. Servicio Nacional de Estudios Territoriales. Balance hídrico integrado y dinamico en El Salvador - Componente evaluacion de recursos hidricos. San Salvador, El Salvador; 2005 p. 118.

10. IARNA-URL, TNC. Bases técnicas para la gestión del agua con visión de largo plazo en la zona metropolitana de Guatemala. Guatemala: The Nature Conservancy - Instituto de Agricultura, Recursos Naturales y Ambiente de la Universidad Rafael Landívar; 2013 p. 124.

11. Lianes Revilla E. Estudio del factor vegetación “factor C” de la Ecuación Universal de Pérdidas de Suelo Revisada “RUSLE” en la cuenca del río Birrís (Costa Rica) [Internet]. Madrid, Spain: Universidad politécnica de Madrid; 2008 p. 191. Available: http://oa.upm.es/1267/

12. Jaramillo Robledo A. La lluvia y el transporte de nutrimentos dentro de ecosistemas de bosque y cafetales. Cenicafé. 2003;54(2): 134–144.

13. Rast W, Lee GF. Nutrient Loading Estimates for Lakes. J Environ Eng. 1983;109: 502–517. doi:10.1061/(ASCE)0733-9372(1983)109:2(502)

14. Jeje Y. Export coefficients for total phosphorus, total nitrogen and total suspended solids in the southern Alberta region [Internet]. Alberta, Canada: Alberta Environment and Sustainable Resource Development; 2006 p. 27. Available: http://environment.gov.ab.ca/info/library/7797.pdf

15. Lin JP. Review of Published Export Coefficient and Event Mean Concentration (EMC) Data [Internet]. Vicksburg, USA: U.S. Army Engineer Research and Development Center; 2004 p. 15. Available: http://el.erdc.usace.army.mil/elpubs/pdf/tnwrap04-3.pdf

16. Hunter HM, Walton RS. Land-use effects on fluxes of suspended sediment, nitrogen and phosphorus from a river catchment of the Great Barrier Reef, Australia. J Hydrol. 2008;356: 131–146. doi:10.1016/j.jhydrol.2008.04.003

17. Johnes PJ. Evaluation and management of the impact of land use change on the nitrogen and phosphorus load delivered to surface waters: the export coefficient modelling approach. J Hydrol. 1996;183: 323–349. doi:10.1016/0022-1694(95)02951-6

18. Fu B, Wang YK, Xu P, Yan K. Modelling nutrient retention function of ecosystem – a case study in Baoxing County, China. Procedia Environ Sci. 2012;13: 111–121. doi:10.1016/j.proenv.2012.01.011

19. Valverde Conejo JC. Riego Y Drenaje. San José, Costa Rica: EUNED; 2007.

20. Burke L, Sugg Z. Modelamiento Hidrológico de la Descarga de las Cuencas Hidrológicas en el Arrecife Mesoamericano [Internet]. Washington, USA: World Resources Institute; 2006 p. 44. Available: https://www.wri.org/sites/default/files/pdf/mar_hydrologic_model_results_spanish.pdf

21. Lianes Revilla E, Marchamalo M, Roldán M. Evaluación del factor C de la RUSLE para el manejo de coberturas vegetales en el control de la erosión en la cuenca del río Birrís, Costa Rica. Agron Costarric. 2009;33: 217–235.

22. Suarez de Castro F, Rodriguez G. Perdidas por erosion de elementos nutritivos bajo diferentes cubiertas vegetales y con varias practicas de conservacion de suelos. Chinchiná, Colombia: Federación Nacional de Cafeteros; 1955 pp. 1–13. Report No.: 2(14).

23. Ramos Taipe CL. Modelamiento ambiental para analisis de susceptibilidad erosiva en la cuenca media y alta del rio Cañete y determinación del mapa de erosión. Lima, Peru: Universidad nacional agraria la Molina; 2001 p. 26.

24. Pinto S, Valerio Filho M, Donzeli P. Soil Erosion Susceptibility Evaluation Based on GIS Technology. International society for Photogrammetry and Remote Sensing. Washington, USA; 1992. pp. 172–174. Available: http://www.isprs.org/proceedings/xxix/congress/part7/172_XXIX-part7.pdf

25. Bengtson RL, Selim HM. USLE “C” Values for Louisiana Sugarcane. Trans ASABE. 2012; doi:10.13031/2013.41712

26. Bengtson RL, Selim HM, Ricaud R. Water quality from sugarcane production on alluvial soils. Trans ASABE. 1998;41: 1331–1336. doi:10.13031/2013.17306

27. Thorburn PJ, Biggs JS, Attard SJ, Kemei J. Environmental impacts of irrigated sugarcane production: Nitrogen lost through runoff and leaching. Agric Ecosyst Environ. 2011;144: 1–12. doi:10.1016/j.agee.2011.08.003

28. Filoso S, Martinelli LA, Williams MR, Lara LB, Krusche A, Ballester MV, et al. Land use and nitrogen export in the Piracicaba River basin, Southeast Brazil. Biogeochemistry. 2003;65: 275–294. doi:10.1023/A:1026259929269

29. Webster AJ, Bartley R, Armour JD, Brodie JE, Thorburn PJ. Reducing dissolved inorganic nitrogen in surface runoff water from sugarcane production systems. Mar Pollut Bull. 2012;65: 128–135. doi:10.1016/j.marpolbul.2012.02.023

30. Kwong KFNK, Bholah A, Volcy L, Pynee K. Nitrogen and phosphorus transport by surface runoff from a silty clay loam soil under sugarcane in the humid tropical environment of Mauritius. Agric Ecosyst Environ. 2002;91: 147–157. doi:10.1016/S0167-8809(01)00237-7

31. Allen RG, Pereira LS, Raes D, Smith M. Crop evapotranspiration-Guidelines for computing crop water requirements. Rome, Italy: FAO; 1998. Report No.: 56.

32. Gutiérrez MV, Meinzer FC. Estimating Water Use and Irrigation Requirements of Coffee in Hawaii. J Am Soc Hortic Sci. 1994;119: 652–657.

33. Avila H, Harmand JM, Dambrine E, Jimenez F, Beer J, Oliver R. Dinamica del nitrogeno en el sistema agroforestal “Coffea arabica” con “Eucalyptus deglupta” en la zona sur de Costa Rica. Agroforesteria En Las Am. 2004;11: 83–91.

34. Esquivel Vargas C. Cuantificación de las Tasas De Evapotranspiración De Seis Coberturas Del Humedal Palo Verde, Parque Nacional Palo Verde, Costa Rica [Internet]. Cartago, Costa Rica: Instituto Tecnológico de Costa Rica - Escuela de ingeniería forestal; 2013 p. 41. Available: http://repositoriotec.tec.ac.cr/handle/2238/3110

35. Kelsey P. Nutrient-export modelling of the Leschenault catchment. Australia: Department of Water, Western Australia; 2010 p. 64. Report No.: 11.

36. Wang X, Feng A, Wang Q, Wu C, Liu Z, Ma Z, et al. Spatial variability of the nutrient balance and related NPSP risk analysis for agro-ecosystems in China in 2010. Agric Ecosyst Environ. 2014;193: 42–52. doi:10.1016/j.agee.2014.04.027

37. Campos Vargas CA. Análisis de los cambios de cobertura de la cuenca alta y media del Río Reventazón, Costa Rica, periodo 2000-2010 [Internet]. Cartago, Costa Rica: Instituto Tecnológico de Costa Rica - Escuela de ingeniería forestal; 2010 p. 83. Available: http://bibliodigital.itcr.ac.cr/xmlui/handle/2238/3002

38. Cifuentes-Jara M. Aboveground Biomass and Ecosystem Carbon Pools in Tropical Secondary Forests Growing in Six Life Zones of Costa Rica. USA: Oregon State University; 2008 p. 195.

39. Ruiz A, Ibrahim M, Locatelli B, Andrade H, Beer J. Fijación y almacenamiento de carbono en sistemas silvopastoriles y competitividad económica de fincas ganaderas en Matiguas, Nicaragua. AgroForestería En Las Américas. 2004; 16–21.

40. Avila Vargas G. Fijación y almacenamiento de carbono en sistema de café bajo sombra,café a pleno sol, sistemas silvopastoriles y pasturas a pleno sol. Turrialba, Costa Rica: CATIE; 2000 p. 116.

41. Rojas J, Ibrahim M, Andrade HJ. Secuestro de carbono y uso de agua en sistemas silvopastoriles con especies maderables nativas en el trópico seco de Costa Rica. Rev Corpoica Cienc Tecnol Agropecuaría. 2009;10: 214–223.

42. Amézquita MC, Murgueitio E, Ibrahim M, Ramírez B. Carbon sequestration in pasture and silvo-pastoral systems under conservation management in four ecosystems of tropical America. FAO/CTIC Conservation Agriculture Carbon Offset Consultation. Food and Agriculture Organization of the United Nations (FAO). Rome, Italy; 2008. pp. 1–11. Available: https://cgspace.cgiar.org/handle/10568/56200

43. Ibrahim M, Chacón M, Cuartas C, Naranjo J, Ponce G, Vega P, et al. Almacenamiento de carbono en el suelo y la biomasa arbórea en sistemas de usos de la tierra en paisajes ganaderos de Colombia, Costa Rica y Nicaragua. Agroforestería En Las Américas. 2007; 27–36.

44. Ramos Veintimilla R. Fraccionamiento del carbono orgánico del suelo en tres tipos de uso de la tierra en fincas ganaderas de San Miguel de Barranca, Puntarenas-Costa Rica [Internet]. Turrialba, Costa Rica: CATIE; 2003 p. 96. Available: http://bibliotecadigital.catie.ac.cr:8080/repositorio/handle/123456789/5156

45. Holmann F, Rivas L, Pérez E, Castro C, Schuetz P, Rodríguez J. La cadena de carne bovina en Costa Rica: Identificación de temas críticos para impulsar su modernización, eficiencia y competitividad [Internet]. Cali, Colombia: Centro Internacional de Agricultura Tropical (CIAT) - Internacional Livestock Research Institute (ILRI) - Corporación Ganadera (Corfoga); 2007 p. 75. Report No.: 206. Available: http://www.corfoga.org/images/public/documentos/pdf/cadena_carne_bovina_CR.pdf

46. Tencio R. Informacion General Region Central Oriental [Internet]. Costa Rica: MAG Región Central Oriental; 2013 p. 14. Available: https://docs.google.com/viewer?a=v&pid=sites&srcid=ZGVmYXVsdGRvbWFpbnxyZWdpb25jZW50cmFsb3JpZW50YWx8Z3g6OTZmMzgwOGIyYjkyMWJj

47. Montenegro Ballestero J, Chaves Solera M. Emisión de gases por la caña de azúcar: propuesta metodológica para realizar un balance de carbono. 2009; San José, Costa Rica.

48. Beeharry RP. Carbon balance of sugarcane bioenergy systems. Biomass Bioenergy. 2001;20: 361–370. doi:10.1016/S0961-9534(00)00094-5

49. Galdos MV, Cerri CC, Cerri CEP. Soil carbon stocks under burned and unburned sugarcane in Brazil. Geoderma. 2009;153: 347–352. doi:10.1016/j.geoderma.2009.08.025

50. Ministerio de Agricultura y Ganadería. Estadísticas Agropecuarias [Internet]. 2012. Available: http://www.mag.go.cr/

51. CEPAL. Subregión norte de América Latina y el Caribe : Información del sector agropecuario: las tendencias alimentarias 2000-2010 [Internet]. México: Comisión Económica para América Latina y el Caribe (CEPAL) - Naciones Unidas; 2011 p. 78. Available: http://repositorio.cepal.org/handle/11362/25461

52. Mena VE, Castañeda HJA, Delgado JM. Biomasa y carbono almacenado en sistemas agroforestales con café y en bosques secundarios en un gradiente altitudinales en Costa Rica. Rev Agroforestería Neotropical. 2011;1: 1–12.

53. IPCC, Prepared by the National Greenhouse Gas Inventories Programme, Eggleston H.S., Buendia L., Miwa K., Ngara T. and Tanabe K. Guidelines for National Greenhouse Gas Inventories [Internet]. Japan; 2006. Available: http://www.ipcc-nggip.iges.or.jp/public/2006gl/index.html

54. Fonseca-González W, Rey Benayas JM, Alice FE. Carbon accumulation in the biomass and soil of different aged secondary forests in the humid tropics of Costa Rica. For Ecol Manag. 2011;262: 1400–1408. doi:10.1016/j.foreco.2011.06.036

55. Fonseca-González W, Alice FE, Montero J, Toruño-Gutiérrez H, Leblanc-Ureña HA. Acumulación de biomasa y carbono en bosques secundarios y plantaciones forestales de Vochysia guatemalensis e Hieronyma alchorneoides en el Caribe de Costa Rica. Agroforestería En Las Américas. 2008; 57–64.
