# Supplementary material for: Dynamics of Ecosystem Services during Forest Transitions in Reventazón, Costa Rica
Source: PLoS One. 2016 Jul 8;11(7):e0158615. doi: 10.1371/journal.pone.0158615 (PMC4938395; doi:10.1371/journal.pone.0158615)

S2. Transformation of ES variables.

To analyze and compare ES evolutions, ES estimated levels were log-transformed (if they had a skewed distribution) and standardized with a Z-score normalization (resulting in values with a mean of 0 and a standard deviation of 1).

Fig 1. Histograms and Q–Q plots of transformed ES variables.

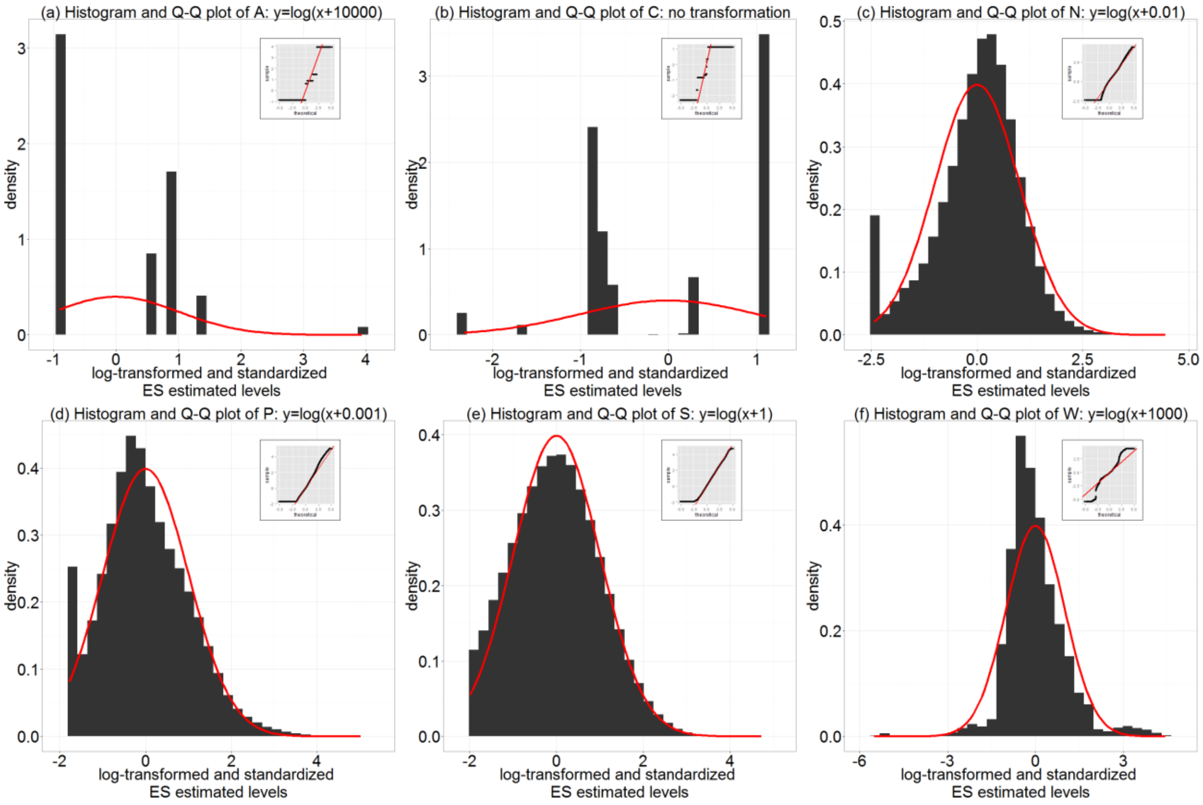

Supplement: S2 File — (PDF) [file pone.0158615.s002.pdf]
