# Supplementary material for: Dynamics of Ecosystem Services during Forest Transitions in Reventazón, Costa Rica
Source: PLoS One. 2016 Jul 8;11(7):e0158615. doi: 10.1371/journal.pone.0158615 (PMC4938395; doi:10.1371/journal.pone.0158615)
Supplement: S3 File — (PDF) [file pone.0158615.s003.pdf]

S3. Details on land-cover changes.

Table 1. Proportion (in %) of each land cover for the four time periods studied (1986, 1996, 2001 and 2008).

Code	Land cover	1986	1996	2001	2008
1	Old forests	39.3	39.5	40.2	40.8
2	Pastures	28.7	27.3	27.3	27.0
3	Young forests	7.4	8.4	7.5	7.4
4	Sugarcane plantations	6.5	6.8	6.7	6.6
5	Coffee plantations	14.7	13.7	13.4	13.2
6	Urban areas	0.7	1.0	1.1	1.1
7	Water bodies	1.8	1.8	2.0	2.0
8	Crops	1.0	1.3	1.3	1.3
9	Bare soil	0.0	0.0	0.0	0.0
10	Forest plantations	0.0	0.0	0.0	0.3
11	Crops under net	0.0	0.0	0.1	0.1
12	Rural areas planned for urbanization	0.0	0.3	0.3	0.3
TOTAL		100	100	100	100
