# Supplementary material for: Dynamics of Ecosystem Services during Forest Transitions in Reventazón, Costa Rica
Source: PLoS One. 2016 Jul 8;11(7):e0158615. doi: 10.1371/journal.pone.0158615 (PMC4938395; doi:10.1371/journal.pone.0158615)
Supplement: S4 File — (PDF) [file pone.0158615.s004.pdf]

S4. Results of the sub-watershed cluster analysis.

To highlight different dynamics within the study area, we defined three groups of sub-watershed based on forest area change from 1986 to 2008: large increase (in more than 3% of the area, a threshold defined arbitrarily as the 80% quantile of the distribution of the absolute values of forest area changes), moderate increase (in less than 3% of the area), and decrease or no change (in less than 3% of the area). To analyze changes in forests area, we considered all forest ecosystems described previously: old, young and planted forests.

Table 1. Mean forest area change (in %) from 1986 to 2008 in each group identified.

**Groups**	**Subwatershed numbers**	**Mean forest area change (in %)**
Decrease or no change	1, 9 and 11	-1.28
Moderate Increase	3, 4, 6, 7, 8, 10, 12 and 13	1.16
Large Increase	2 and 5	10.49

The k-means algorithm was used to cluster the 13 sub-watersheds according to the changes of ES observed in these sub-watersheds between 1986 and 2008.

Table 2: Mean changes in the levels of the six selected ES from 1986 to 2008 in each cluster identified (A: agricultural production, C: carbon, N: nitrogen retention, P: phosphorus retention, S: sediment retention, W: water yield).

**Clusters**	**Subwatershed numbers**	**A**	**C**	**N**	**P**	**S**	**W**
Weak tradeoffs: more food, less carbon and water	1, 7 and 9	0.020	-0.036	0.203	0.157	0.004	-0.020
Weak tradeoffs: more carbon, less food	3, 4, 6, 8, 10, 11, 12 and 13	-0.019	0.017	0.214	0.135	-0.009	-0.004
Strong tradeoffs: more carbon, less food	2 and 5	-0.156	0.120	0.206	0.102	-0.030	0.004
