# Supplementary material for: Dynamics of Ecosystem Services during Forest Transitions in Reventazón, Costa Rica
Source: PLoS One. 2016 Jul 8;11(7):e0158615. doi: 10.1371/journal.pone.0158615 (PMC4938395; doi:10.1371/journal.pone.0158615)
Supplement: S5 File — (PDF) [file pone.0158615.s005.pdf]

S5. Linear models of land-cover changes.

We developed linear models to analyze the effects of four explanatory variables (distance to human settlements, distance to roads, terrain slope and altitude) on land-cover changes at the resolution of 30m pixels. We analyzed six major land-cover changes observed between 1986 and 2008 in the study site:

- forest degradation (from old to young forests),
- expansion of agriculture (pastures, coffee and crops) over old or young forests,
- forest regeneration (from young to old forests),
- abandonment of agricultural lands (coffee plantations and pastures) followed by young forests,
- shift in agricultural production (e.g. coffee to horticulture, pasture to sugarcane),
- urbanization.

For each type of land-cover change, we selected a sample of 500 pixels (250 having experienced the change and 250 having not) and built a logistic regression model. The sampling was done to avoid the effect of very large samples (our dataset had more than 750,000 pixels), which lead to very low p-values and overestimate the significance of explanatory variables [1,2]. The logistic regression model predicted a binary variable of land-cover change (1 if the type of land-cover change occurred, 0 otherwise). We evaluated the performance of the model by assessing how it predicted land-cover change using a new random set of data (fitted values below 0.5 were considered as predicting no land-cover change, and above 0.5 as predicting land-cover change). The accuracy was calculated as the percentage of correctly predicted pixels. Because results may be sensitive to the randomization processes, we ran 100 iterations of model building and evaluation for each type of land-cover changes and we reported the range of accuracy values and median p-values (Table 1).

Results showed that urbanization took place close to human settlements, while changes between forest and agriculture (forest degradation, expansion of agricultural lands, forest regeneration, and abandonment of agricultural lands) occurred further away (Table 1). Similarly urbanization, abandonment of agricultural lands and shifts in agricultural production occurred close to roads, while forest degradation took place further from roads. Forest regeneration occurred more on steep slopes, while shifts in agricultural production and urbanization happened more in flat areas. Finally, the model showed that low altitude areas experienced much more land-cover changes (including forest degradation, abandonment of agricultural lands, shifts in agricultural production, and urbanization) than high altitude areas.

Table 1. Results of logit models

**Land-cover change**	**Accuracy range over the iterations**	**Significant and effect of the predictors (in parenthesis): *** (median p over all iterations >0.001), ** (<0.01), * (<0.1), NS (other significant), + (positive effect), - (negative effect)**			
		**Distance to human settlements**	**Distance to roads**	**Slope**	**Altitude**
Forest degradation	74-82%	** (+)	*** (+)	NS	*** (-)
Expansion of agricultural lands	56-71%	*** (+)	NS	NS	NS
Forest regeneration	50-63%	*** (+)	NS	* (+)	NS
Abandonment of agricultural lands	53-61%	*** (+)	*** (-)	NS	* (-)
Shifts in agricultural production	64-75%	NS	*** (-)	*** (-)	* (-)
Urbanization	76-86%	* (-)	*** (-)	* (-)	*** (-)

References

1. Lin M, Lucas HCJ, Shmueli G. Too Big to Fail: Large Samples and the p-Value Problem. Inf Syst Res. 2013;24: 906–917. doi:10.1287/isre.2013.0480

2. Sullivan GM, Feinn R. Using Effect Size - or Why the P Value Is Not Enough. J Grad Med Educ. 2012;4: 279–282. doi:10.4300/JGME-D-12-00156.1
